# Multi-Section Sensing and Vibrotactile Perception for Walking Guide of Visually Impaired Person

**DOI:** 10.3390/s16071070

**Published:** 2016-07-12

**Authors:** Gu-Young Jeong, Kee-Ho Yu

**Affiliations:** 1 Center for Healthcare Technology Development, Chonbuk National University, Jeonju 54896, Korea; jung902@chonbuk.ac.kr; 2 Department of Aerospace Engineering, Chonbuk National University, Jeonju 54896, Korea

**Keywords:** Electronic Travel Aid, obstacle detection, vibrotactile, tactile display, visually-impaired

## Abstract

Electronic Travel Aids (ETAs) improve the mobility of visually-impaired persons, but it is not easy to develop an ETA satisfying all the factors needed for reliable object detection, effective notification, and actual usability. In this study, the authors developed an easy-to-use ETA having the function of reliable object detection and its successful feedback to the user by tactile stimulation. Seven ultrasonic sensors facing in different directions detect obstacles in the walking path, while vibrators in the tactile display stimulate the hand according to the distribution of obstacles. The detection of ground drop-offs activates the electromagnetic brakes linked to the rear wheels. To verify the feasibility of the developed ETA in the outdoor environment, walking tests by blind participants were performed, and the evaluation of safety to ground drop-offs was carried out. From the experiment, the feasibility of the developed ETA was shown to be sufficient if the sensor ranges for hanging obstacle detection is improved and learning time is provided for the ETA. Finally, the light-weight and low cost ETA designed and assembled based on the evaluation of the developed ETA is introduced to show the improvement of portability and usability, and is compared with the previously developed ETAs.

## 1. Introduction

To a visually-impaired person, it is very important to obtain spatial information, including the locations of obstacles and walkways for moving safely; hence, they have used white canes as mobility tools—a long, white cane is “traditional” and the primary mobility tool. An Electronic Travel Aid (ETA) is a device that collects environmental information and transmits it to the visually-impaired user to allow independent movement. Generally, ETAs detect obstacles in the user’s walking paths through the use of more than one sensor and provide the user with information on the obstacle distribution with various notification methods: sounds, vibrations, etc.

Improving the performance of ETAs is closely connected with enhancing the mobility of visually-impaired people, and therefore, many researchers are trying to develop technology for a more practical and effective ETA. Benjamin et al. [1] developed a Laser Cane, which is a conventionally-shaped long cane that sends out three laser beams to detect overhead obstacles, obstacles at waist level, and drop-offs. The Laser Cane gives auditory and tactile warnings when it detects obstacles. A laser beam is employed by the Laser Cane, but ultrasound is commonly used in most ETAs [2]. Sonic Torch (Kay, 1964) [3], Nottingham Obstacle Detector (Armstrong, 1973) [4], Mowat sensor (Pressey, 1977) [5], and UltracaneTM [6] are examples of hand-held devices using ultrasonic sensors. Sonic Torch and Nottingham Obstacle Detector provide auditory output, whereas Mowat sensor and UltracaneTM have tactile output. Visually-impaired people can detect obstacles and potential obstacles on the walking path by swinging the devices. Russell Pathsounder (Russell, 1965) [7], Sonic Pathfinder (Dodds, 1984) [8], and Navbelt (Shoval, et al. 1994) [9] are wearable ETAs that utilize ultrasonic sensors. The Russell Pathsounder is suspended from the user’s neck and detects obstacles in the front of the user; a beeping sound and vibration are used as warning notifications. Sonic Pathfinder is a head-mounted device designed to allow discrimination of obstacle direction while basically remaining an obstacle detection system. Obstacle detection is performed by five ultrasonic transducers, and the obstacle direction is fed to one of the two ear pieces, depending on its location. Navbelt, worn around the waist, creates a map of the angle and the distance of any obstacle using eight ultrasonic sensors and provides acoustic feedback. GuideCane (Ulrich and Borenstein, 2001) [10] is the second project by Borenstein, and is a device similar to a white cane, guiding the walking direction via tactile feedback to avoid the detected obstacle. In addition, the EPFL prototype (Cardin, et al. 2007) [11] is a wearable system that detects obstacles by the shoulder-mounted sonar system and informs the user about its localization via vibrotactile feedback. Vision-based ETAs which employ a camera as a sensor have recently been developed with powerful microprocessors. The vOICe (Meijer, 1992) [12] captures images of the environment and directly maps visual attributes such as brightness and color to aural attributes such as tone, pitch, and loudness. NAVI (Nagarajan, et al. 2003) [13] identifies objects in the image using image processing techniques with fuzzy logic and sends the object identification results to the user through the headphones. Both vOICe and NAVI are single-camera systems with no depth information on objects, whereas ENVS (Meers and Ward, 2004) [14] extracts a depth map from two cameras to deliver environmental depth information to the fingers using a tactile stimulator. Tyflo (Dakopoulos, et al. 2007) [15,16] consists of two miniature cameras, a microphone, an ear speaker, the 2D vibration array, and a portable computer. The cameras capture images from the surrounding environment, and the processed 3D space representations are projected on the 2D array vibrators attached to the user’s chest.

Dakopoulos et al. [16] surveyed and evaluated the existing 22 ETAs for the blind, including some of the ETAs mentioned above. In the paper, they pointed out that the design of a proper interface between the system and the user for appropriate information transfer to the user with a robust interaction scheme is the most challenging issue. Velazquez [17] reviewed the wearable technologies and devices to assist the blind. Schinazi et al. [18] reviewed many topics on spatial navigation by congenitally blind persons, with an emphasis on behavioral and neuroscientific evidence, as well as the potential of technological assistance. Regarding recent research, a tactile feedback-assisted virtual cane system operated by a finger pointing gesture [19], an indoor navigation system using Ultra-Wideband (UWB) technology [20], and a virtual blind cane system consisting of a camera, line laser, and an inertial measurement unit for indoor application [21] were reported.

The correct walking plan of the user depends on the exact delivery of obstacle distribution to the user. Therefore, the notification method of obstacle distribution is as important as the obstacle detection method. The use of sensory substitution is an alternative way to transfer visual information of an obstacle and walking environment to the blind user. Some of the sensory substitution devices (SSD)—such as tongue display unit (TDU) [22] and auditory and tactile feedback [23]—were proposed and tested for a walking guide of the blind. Stronks et al. [24] reviews the studies of the influence of training and visual deprivation on the performance of various sensory substitution approaches. The ETAs based on acoustic devices allows the provision of various information about the guide way or surroundings, but it can place a visually-impaired person in a grave situation because a significant sound may be covered by another one [16,25]. Excessive concentration on the acoustic signals from an ETA may reduce the ability of the visually-impaired to hear these essential signs. The information delivered from ETAs based on tactile display does not occupy the auditory sense, so unexpected interference in the recognition of information is no longer an issue. Many other techniques associated with tactile feedback may be used to tactile notification [26,27]. However, in ETAs based on tactile display, the important thing to take into consideration is how well visually-impaired people can perceive the stimulus from the tactile display. This is associated with the sensitivity of the human tactile sense and the effectiveness of the tactile display. It is not easy to develop an ETA satisfying all factors needed for reliable object and/or obstacle detection, effective notification, and actual usability. During the last decades, various ETAs for the visually-impaired person have been developed, and some of them were provided as marketable products. However, the products have limited functionalities and high cost, and other developed ETAs showed insufficient maturity—especially in the features of reliability and performance [16,17]. Therefore, reliable detection of objects and sufficient performance for transfer of object information and obstacle avoidance are strongly required. Excellent usability and low cost are also recommended to assist visually-impaired people actually and broadly in their daily life.

In the present study, the authors developed an easy-to-use and low cost ETA having the function of reliable object detection and its successful feedback to the user by tactile stimulation. To achieve this goal, a guide vehicle including multiple ultrasonic sensors, electromagnetic brakes, and tactile display was developed. Multiple ultrasonic sensors face in different directions to scan the surroundings, while the electromagnetic brakes ensure the safety in down-step. The tactile display was designed to make sure of the accordance between the detected obstacle’s position and the location of the perceived obstacle.

## 2. System Configuration

**Figure 1:**
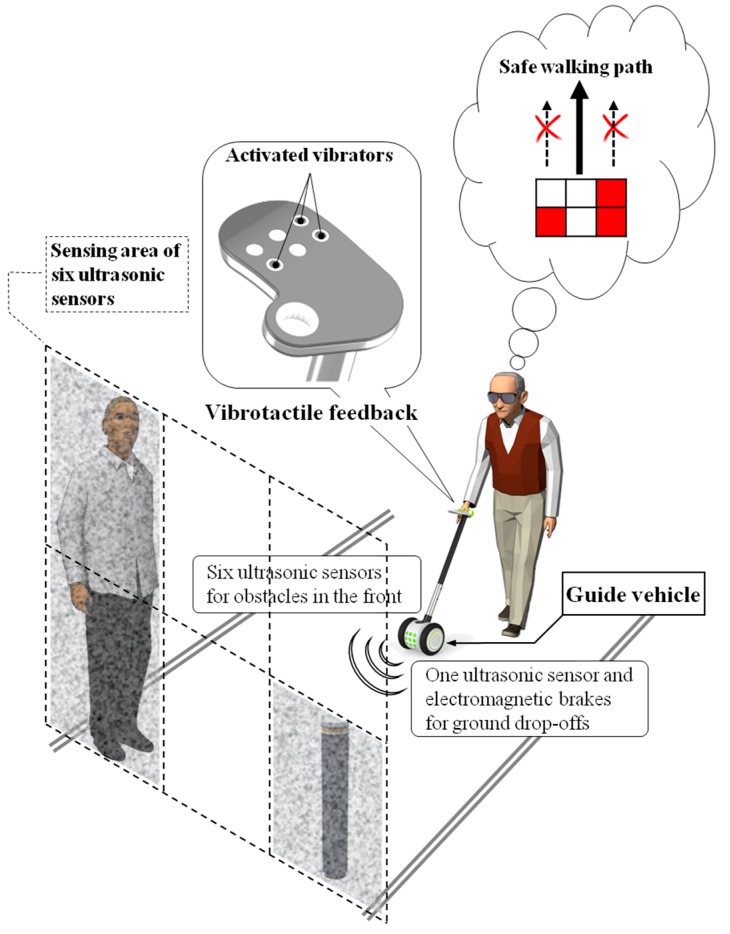
Concept of the guide device with tactile feedback and multi-ultrasonic sensors.

**Figure 2:**
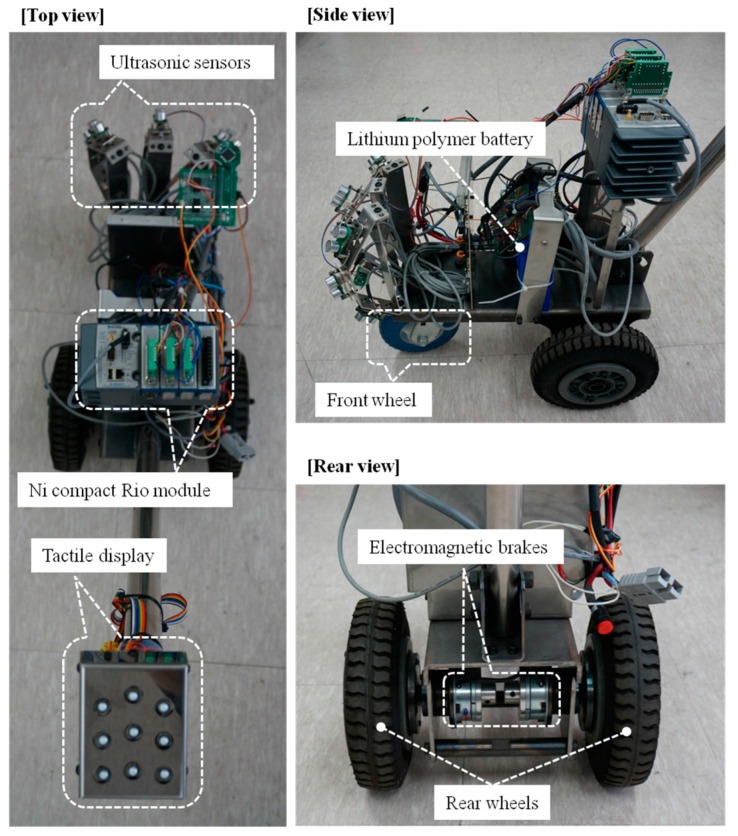
The developed Electronic Travel Aid (ETA) system.

The ETA developed in this study consists of an obstacle detection unit, a tactile display, electromagnetic brakes and a control unit. Seven ultrasonic sensors were placed facing in different directions to detect obstacles in the walking path, and vibrators in tactile display stimulate the hand according to the obstacle distribution, as shown in Figure 1. The concept of GuideCane [10] and EPFL [11] are similar to the developed system. The ultrasonic sensors of GuideCane detect an obstacle, the embedded computer determines a suitable walking direction, and then steers the GuideCane. The EPFL detects an obstacle by using a multi-sonar system and feeds back appropriate vibrotactile output to the blind user. However, those systems have limitations in scanning area and object detection. The proposed system was designed to overcome these limitations. The space in front of the guide vehicle is divided into six sections, each of which is monitored by one of six ultrasonic sensors. In these sections, each column represents objects in the azimuth direction. The upper rows represent hanging and/or tall obstacles, while the bottom rows represent objects on the ground. Especially the detection of head-height obstacles, not only on the ground, is important for the walking blind person to secure free walking space [16], and the sensors of upper rows are used to carry out this function. The six ultrasonic sensors are connected to six vibrators in the tactile display, which contacts to the fingers consisting of six phalanges corresponding to two by three tactile elements. The adoption of the fingers for transfer of tactile stimulation improves the perception performance of vibrotactile feedback compared to the palm [28]. The user of the guide vehicle realizes the presence of obstacles and makes an obstacle avoidance plan by recognizing the vibrotactile feedback. The remaining one ultrasonic sensor looks down at the ground for the detection of ground drop-offs, and activates the electromagnetic brakes linked to the rear wheels. The wearable-type navigation device has the advantage of portability and free hand; on the other hand, the vehicle-type navigation system proposed in this paper provides buffer space between the user and an unexpected obstacle, even if the obstacle detection fails. Additionally, the sensed information from the vehicle is relatively robust compared to the wearable device, because the sensors attached to the front of the vehicle are relatively free of the motion artifact of the user due to walking movements. All of the electronic parts are controlled by a compact-RIO module (National Instrument Inc., Austin, TX, USA), which was programmed with LabVIEW software. The capacity of the used Li-Po battery is 9000 mAh, and the continuous running time is 5 h in case of full operation of all electrical parts. The developed ETA is shown in Figure 2.

### 2.1. Detection of Obstacle Distribution

**Figure 3:**
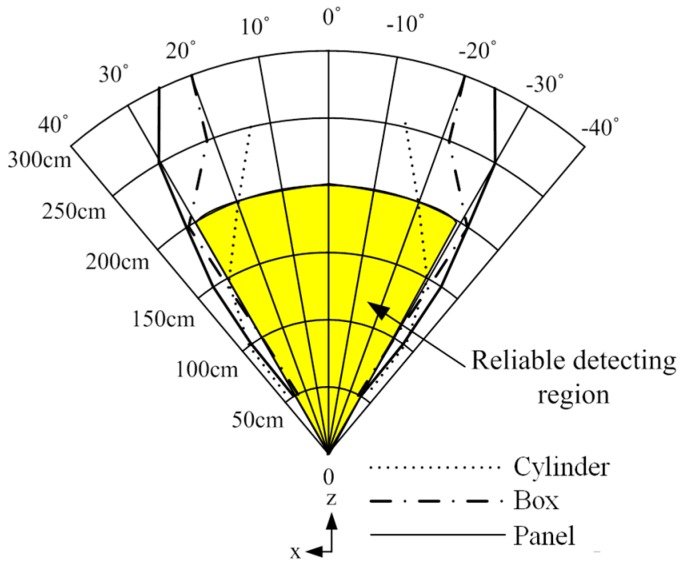
Detection region of one ultrasonic sensor (Each line represents the detectable region of the object, respectively).

Ultrasonic sensors (SRF05, Devantech Co. Ltd., Attleborough, UK) were used to detect obstacle distribution in the walking paths of the visually-impaired. To arrange the ultrasonic sensors properly, estimation of the detecting region of the ultrasonic sensor was carried out. In this experiment, one ultrasonic sensor and three types of objects were used: cylinder type (8 cm diameter, 85 cm height), box type (40 cm width, 125 cm height) and panel type (180 cm width, 145 cm height). The objects used in the experiment represent various obstacles that the visually-impaired encounter in walking paths—for example, blocking rod (cylinder type), pillar or human being (box type), and wall or wide obstacles (panel type). Similar type of obstacles were also used to test some of the sensory substitution devices or ETAs, and Chebat, et al. [22] clarified that obstacle size and avoidance strategy had a significant effect on performance. The detectable range of the ultrasonic sensor was measured by changing the distances between the sensor and objects as follows: 50 cm, 100 cm, 150 cm, 200 cm, 250 cm, and 300 cm. At each distance, the detectable angles were estimated by moving the obstacle outside from the center of the sensor until the obstacle was no longer detected. The detection region of the ultrasonic sensor obtained through experimentation is shown in Figure 3. From the results, the reliable detection region was found to be within 200 cm and ±30 degrees. The measurement errors of distances were under 2 cm.

**Figure 4:**
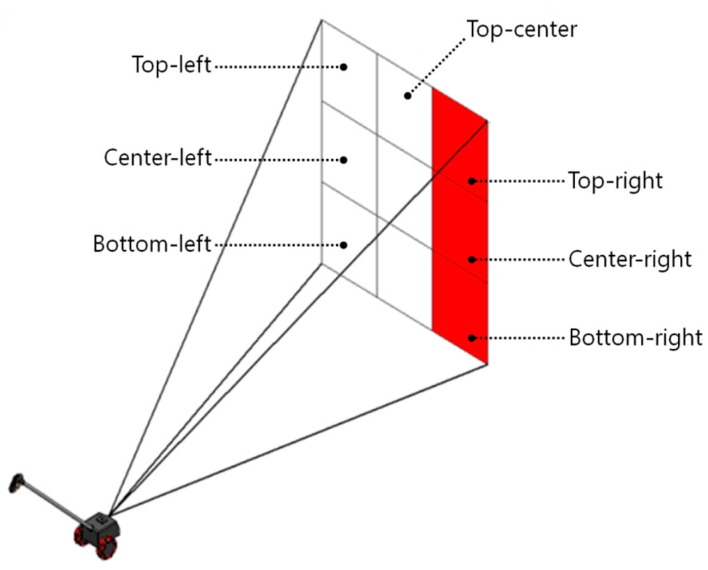
Division of the front area.

A simple way to describe a specific location in front of the eyes is to use the appropriate words, such as ”center”, ”right”, ”left”, ”top-left”, or ”bottom-right”. If we divide the space in front of the eyes into three by three grids, as illustrated in Figure 4, each element grid can be expressed by the words previously mentioned. This space partitioning is a great help in arranging ultrasonic sensors and designing the tactile display. The ETA developed by Zelek et al. [29] utilizes a tactile glove with five piezoelectric actuators on each fingertip corresponding to a spatial direction: more left, a little left, straight ahead, a little right, and more right. The major advantage of this notification method is that the obstacle distribution can be known intuitively. The ETA herein has a more intuitive tactile feedback system because it is obvious to classify an obstacle’s position by dividing the front space into right, center, and left.

**Figure 5:**
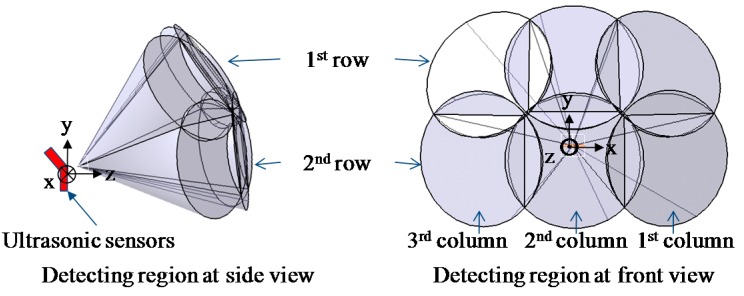
Sensing area of the six ultrasonic sensors.

**Figure 6:**
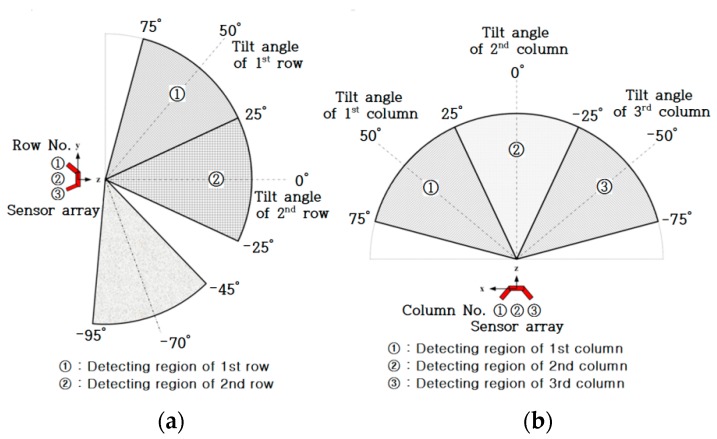
Arrangement of the seven ultrasonic sensors; (**a**) Elevation direction; (**b**) Azimuth direction.

Figure 5 shows the concept of the detection of obstacle distribution using six ultrasonic sensors. In this study, to improve user recognition of obstacle positions, the front space of the user was divided into six regions, with each ultrasonic sensor detecting the assigned region. Seven ultrasonic sensors were used to develop the ETA, the arrangement making a three by three array of ultrasonic sensors. Figure 6a represents the detection region of each row sensor in the array. The first and second row of sensors in the sensor array detects the obstacle distribution on the ground; the sensors in the first row distinguish hanging or tall obstacles from those on the ground. The final sensor in the third row detects ground drop-offs by monitoring ground level. The sensors in the first and second row are connected to a tactile feedback system, while the sensor in the third row is connected to the electromagnetic brake system without providing tactile feedback. The detection region for each sensor was determined to be 50° (±25°) considering the reliable detection region as shown in Figure 3, deduced from the preliminary experimental results. Resultantly, the overlap of 5° occurs with the neighboring sensor, making it difficult to identify the exact position of the obstacles in the overlap region. Nevertheless, such a sensor arrangement contributes to safe and complete detection of obstacles without missing any obstacles in the boundaries of the adjacent sensors. For the above reason, the tilt angle of the first and third row of sensors was 50° and −70°, respectively. In Figure 6b, the detection region in the azimuth direction was 150°, and the detection region for each sensor was determined to be 50° for the same reason.

**Figure 7:**
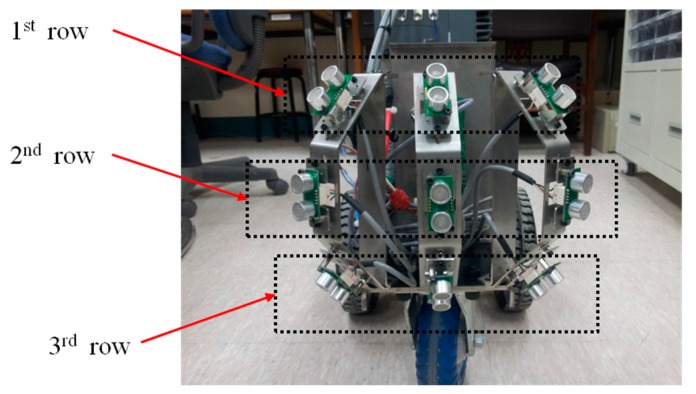
Arrangement of sensors in the developed ETA.

The tilt angle of the first column of sensors was 50°, the second was 0°, and the third was −50°. The three by three array of ultrasonic sensors in the developed ETA is shown in Figure 7. The sensing distance was set up to 200 cm, considering the reliable detection region deduced from the preliminary experimental results.

**Figure 8:**
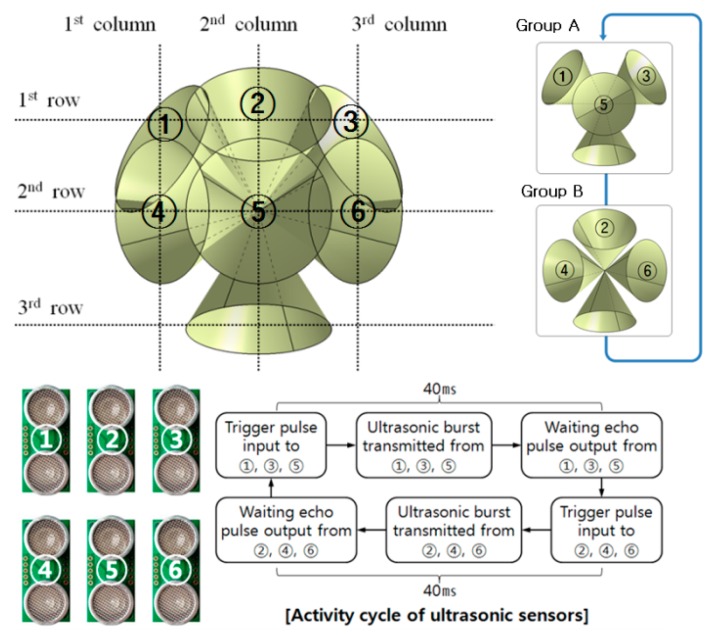
Processing of sensor groups and scanning area.

To update obstacle distribution as soon as possible, the six ultrasonic sensors facing the front were divided into two groups of three sensors. The ultrasound beams of the sensors in a group don’t overlap each other, and all sensors in the group can be activated at the same time. Two groups take turns scanning the obstacles in the front. The process of obstacle detection is represented in Figure 8. It takes 80 ms to scan all of the obstacles in the front space, so that the obstacle detection is performed 12.5 times a second. The detection of ground drop-offs is carried out twenty times in a second. Using the two by three array of ultrasonic sensors, the authors performed a preliminary study on the detection of several kinds of objects in an indoor environment to confirm the usability of the sensor arrangement.

### 2.2. Tactile Display

Effective delivery of obstacle distribution is one of the key factors for an easy-to-use ETA, and it is positively necessary that the position of perceived obstacles is in good accordance with the location of detected obstacles. Hence, a three by three array of ultrasonic sensors was applied to the design of the tactile display.

**Figure 9:**
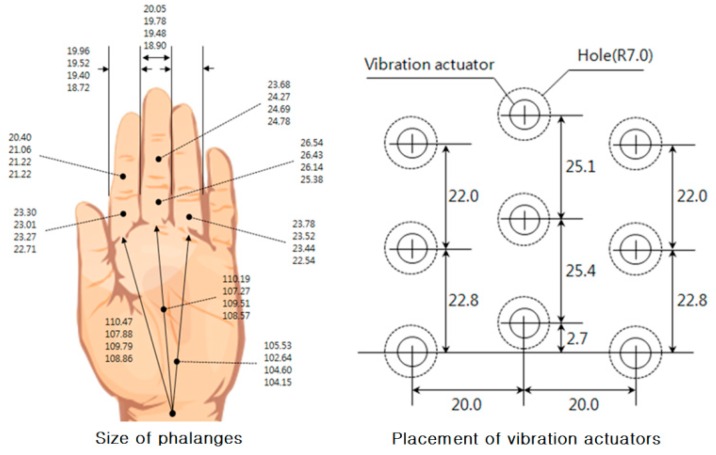
Arrangement of vibrotactile actuators (unit: mm).

In this study, tactile stimulators with nine vibration actuators were developed, and the tactile display stimulates the area of the fingers, which consists of nine phalanges: the first, second, and third phalange of the index, middle, and ring finger. This area has a three by three matrix and will be called the fingers. The space in front of the user was divided into three by three grids. Therefore, a three by three tactile stimulator is suitable to match the partitioned spaces with the vibration actuators. That is, the user of the ETA is able to intuitively know where an obstacle is from the tactile stimulation. Vibration motors were placed 20 mm apart to satisfy the two-point resolution of the palm (about 11 mm). The sizes of phalanges recorded in the database of the Korean Agency for Technology and Standards was referenced to determine the dimensions of the vibration actuators, arranged as shown in Figure 9. This arrangement is favorable for the transmission of a vibratory stimulus to the fingers by matching one phalange with one vibration actuator in the event of obstacle detection. In case of right-handed users, the first phalange of the index finger is put on the vibration actuator of the left-top, and other phalanges are put on the corresponding placement of the vibration actuator. As a result, if an obstacle is detected by the left-top sensor regarding the position of the user, the first phalange of the index finger of the right hand is stimulated by the corresponding actuator. It was confirmed through preliminary experiments that the vibrotactile perception on the fingers was superior to that on the palm [28].

**Figure 10:**
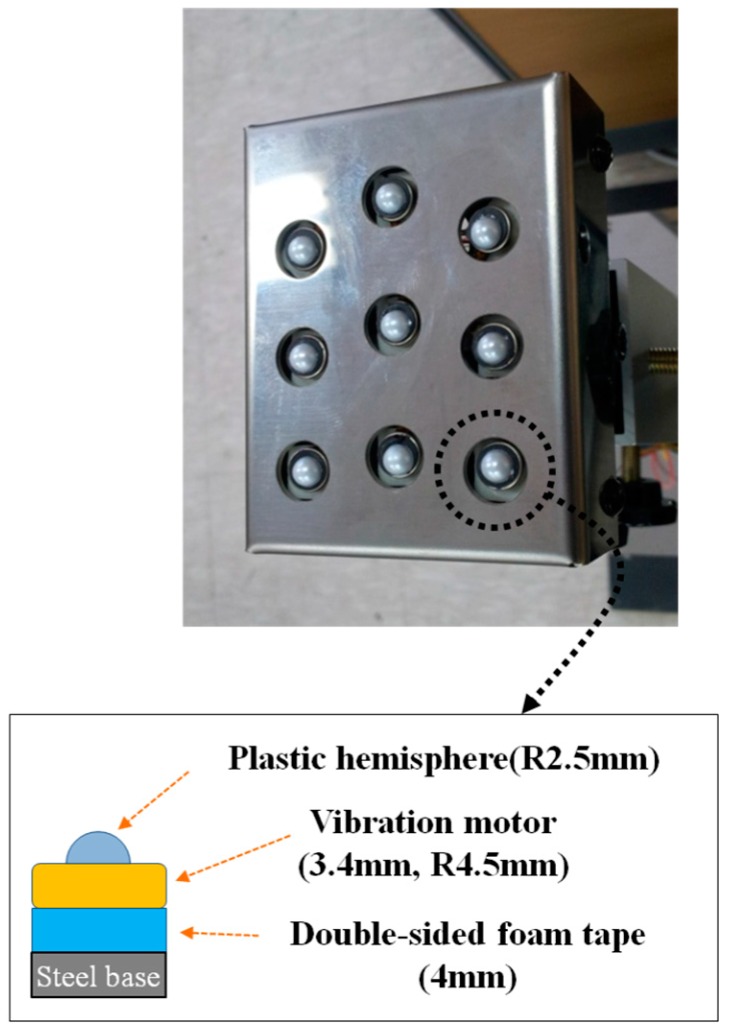
Configuration of the tactile display.

There are many kinds of vibration actuators, but the vibration motor that has often been used recently in research on human tactile sense and tactile display was selected because its intensity and frequency can be easily controlled by adjusting the input voltage, and it can activate many tactile senses simultaneously. Coin-type vibration motors (MB-1004V, Motorbank Inc., Seoul, Korea) were used for the actuators of the tactile stimulators. The size (diameter × height) was 9.0 mm × 3.4 mm. The vibrotactile localization of the vibration motor with a small diameter was reported to be better than the vibrotactile localization of one with a large diameter under conditions using the same actuator spacing, frequency, and intensity [30,31]. Therefore, the smallest coin-type vibration motor that could be found was utilized. To prevent the vibration from spreading from the activated vibration motor to non-activated vibration motors via the steel frame, double-sided foam tape was used to attach the vibration motors to the base frame. The two-point resolution on the palm was reported as 11 mm [32]. A sufficient margin of two-point resolution on the palm was secured by putting a plastic hemisphere on the vibration motors, as shown in Figure 10. The developed tactile display was a three by three tactile stimulator representing obstacle distribution in the front. The vibration motors for the third phalanges—representing ground drop-offs—were not used, because the detection of ground drop-offs instead activated the electromagnetic brakes without any vibrotactile notification. That is, the developed tactile display works as a two by three tactile stimulator for feedback about obstacles on the ground.

The amplitude and frequency of vibrotactile stimuli were fixed to about 100 µm and 96.2 Hz based on the maximum amplitude of the vibration motor used in the developed tactile display, the configuration of which is shown in Figure 10. While walking, the blind user pushes and steers the vehicle by the hand while the fingers are put on the corresponding actuator to keep close contact. Vibratory actuators have been activated at the frequency range of 100 Hz to 250 Hz in most tactile stimulators. In the case of the intensity of vibrotactile stimulation, amplitudes have ranged from tens of micrometers to hundreds of micrometers, depending on the body site. Yu et al. evaluated the vibrotactile pattern perception on the fingertip and palm using several tactile stimulation patterns while varying the amplitude and frequency [30,33]. Lim et al. measured the skin perception threshold of vibrotactile stimuli at the fingertip varying the frequency from 6.31 Hz to 398.1 Hz [34]. From the study of Lim et al., the amplitude of 100 µm and the frequency of 96.2 Hz were considered to be the stimulation conditions to satisfy the vibrotactile threshold of detection at the finger parts. The vibration characteristics of the vibration motors herein were measured using the laser displacement sensor (LK-3100, KEYENCE Co.).

To evaluate the developed tactile display, an experiment of vibrotactile pattern perception was performed by the subjects [28]. The correct rate of the perception on the fingers was 86.78%, and this result was superior to that on the palm (80.16%). Considering that no information of vibrotactile patterns (including the number of activated tactile elements) was provided for the subjects, the perception rate on the fingers is reasonable and useful to implement the developed device for tactile display.

### 2.3. Obstacle Notification

Six ultrasonic sensors for detecting obstacles on the ground were matched with six vibration actuators, respectively. Each ultrasonic sensor observes the area specified by its setting angle, and the vibration motor is activated when an obstacle is detected in the specified area. In other words, the tactile feedback system makes a map of the obstacle distribution to the vibration actuators. Vibration intensity changes according to the distance between the obstacle and the walking guide vehicle; the closer the obstacle, the stronger the vibration intensity. The vibration intensity is controlled by modulating the pulse width (PWM) applied to the vibration motor. Table 1 shows the duty ratio of the motor control signal corresponding to the distance between an obstacle and the ETA. The duty rate is changed by three levels like a step change, because some minimum quantity of stimulation difference is required for human touch perception [32]. Therefore, the step change of stimulation has the advantage of perceiving the tactile stimulation rather than its gradual change. The user can perceive the distance between the obstacle and the walking guide vehicle as relatively far, intermediate, and near distance approaching to an obstacle.

The ultrasonic sensor pointing downward has no connection with the tactile display. It is used only to operate the electromagnetic brake. The downward-facing sensor scans for ground drop-offs as quickly as possible, and the control unit of the ETA puts that sensor’s event before everything else.

## 3. Experiments

This study included a real walking test for blind people for the evaluation of the developed ETA in a life-size obstacle course. The obstacles were made to represent the objects that can be realistically encountered in a walking environment, such as box type object, post or blocking rod, crossing bar, and hanging object. The focus of the walking test was to verify feasibility of the ETA for use in the outdoor environment through the evaluation of obstacle avoidance, walking speed with the ETA, and user comments. The walking test was performed three times.

**Figure 11:**
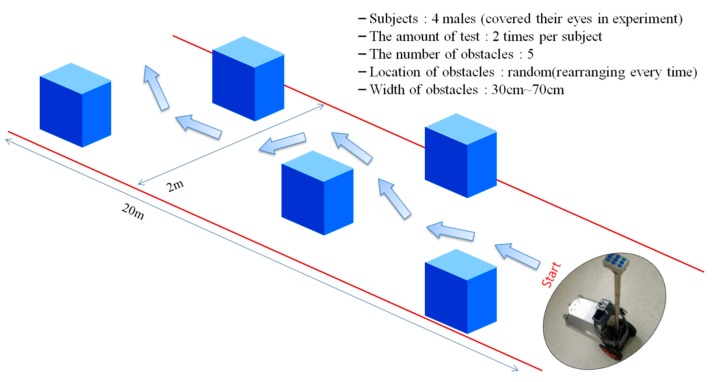
Environmental setup for the first walking test.

The first walking test was done to ensure the safety of walking with the ETA before performing a walking test in an outdoor environment. Four sighted male students (average age: 24.3) participated in the first walking test. The experimental environment was set up indoors as shown in Figure 11, and five cardboard boxes (of different sizes with a width and height of 30–70 cm) were used as obstacles. All participants were blindfolded and tried the walking test twice. The obstacle positions were changed every try.

**Figure 12:**
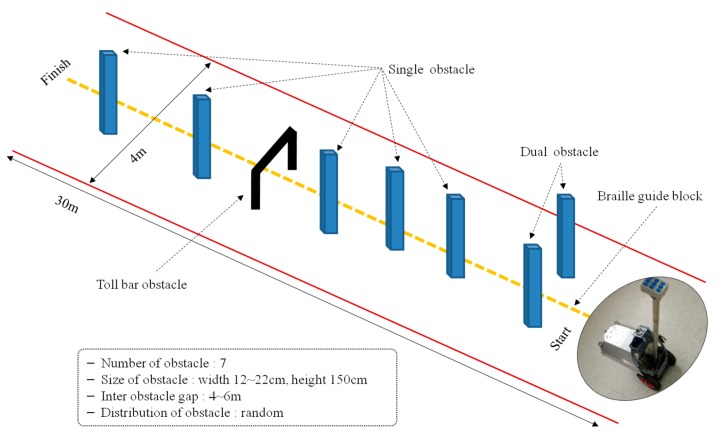
Environmental setup for the second walking test.

The second walking test was carried out after confirming the safety and effectuality of the experimental setup through the first walking test. The experimental environment was set up outdoors, as shown in Figure 12, and five totally blind people (male, average age: 28.9) in their 10 s, 30 s, and 40 s—students of blind school—participated in the test. They are acquired blind participants and use white cane to navigate in their daily life. Additionally, all of them are right-handed users. Only a brief description for mapping vibrotactile stimuli to obstacles’ distribution was provided before the walking test. Braille guiding blocks were set up in the center of the walking path to help the blind participants walk, and experimental conditions similar to a real pedestrian walkway were set up by placing various types of obstacles in the walking path. Five obstacles of single column type (width of 12–22 cm, height of 150 cm), one obstacle of dual column type (distance between columns: 90 cm) and one obstacle of toll bar type (width of 90 cm, height of 80 cm) were placed in the walking path. The height of obstacles was determined based on obstacles in real life and the first walking test included the lower obstacles (30–70 cm). All blind participants completed the walking test three times, and the positions of obstacles were changed every time.

**Figure 13:**
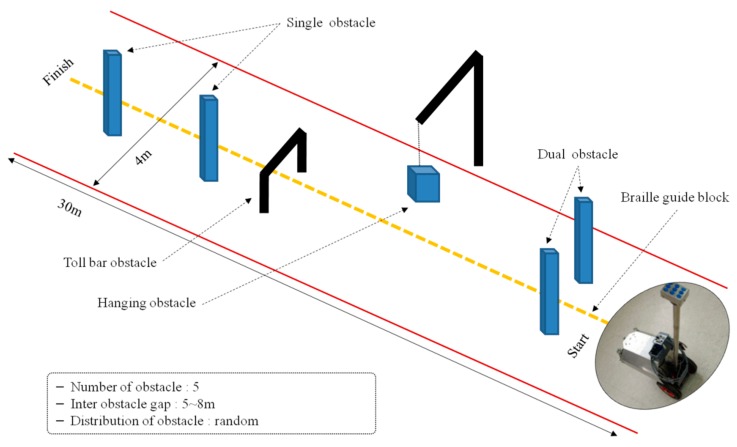
Environmental setup for the third walking test.

For the third walking test, a hanging obstacle—a 24 cm diameter ball—was added as shown in Figure 13. To increase the sections for walking straight, the gap between obstacles was widened by reducing the number of obstacles to five. The sections in which the blind participants could walk straight were shorter in the experimental environment for the second walking test. Five totally blind persons—students of blind school—participated in the third walking test, and they were the same participants of the second walking test. All participants tried the walking test three times, and the positions of the obstacles were changed for every trial, as in the previous walking tests.

**Figure 14:**
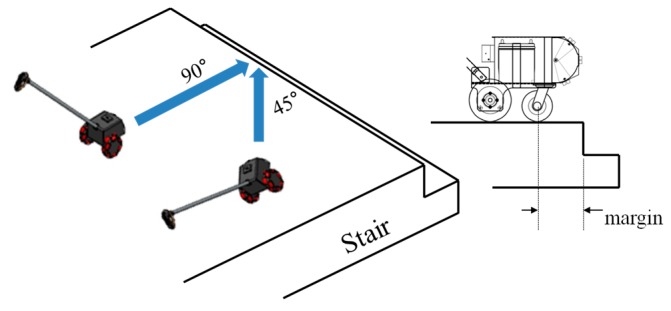
Experimental configuration for testing the braking system.

The test of the avoidance of ground drop-offs was performed indoors without the inclusion of a participant for safety reasons. The braking performance of the developed ETA was evaluated, assuming the situations in which the developed ETA was approaching stairs, as shown in Figure 14.

## 4. Results and Discussion

A walking test for blind people is very effective for evaluating the overall performance of ETAs, because blind people are the real users of ETAs. In this sense, the experimental scenarios herein were very appropriate for the verification of the feasibility of the newly developed ETA.

The results of the first walking test are shown in Table 2. The participants, blindfolded university students, had only five minutes to adjust to the operation method and tactile feedback of the developed ETA before the walking tests. Nevertheless, obstacle avoidance was 90% successful. This result was good enough, because the first walking test was performed to ensure the safety of the developed ETA and the effectuality of the experimental scenario. The four failures in the first walking test occurred in the process of the avoidance operation after the recognition of an obstacle. There were no failures in obstacle detection of the ETA. The second and third walking tests were the major experiments to evaluate the feasibility of the developed ETA. We checked how long it took the blind participants to complete the mission, and wrote down each participant’s collision-free path according to the type and position of obstacles. Unlike in the first walking test, in the second and third walking tests, no time was given to the participants to become familiar with the ETA, but a brief description about the accordance between the location of vibrotactile stimuli and the location of detected obstacles was provided.

The results of the second walking test are shown in Table 3, which itemizes the number of failures and obstacle types that caused them. The rate of successful obstacle avoidance was approximately 88.6%. Avoidance of single obstacles was perfect, while dual and toll bar obstacles achieved about 60%. The process of the avoidance of single obstacles gave ideal results in accordance with our goal—an ETA satisfying all factors for reliable obstacle detection, effective notification, and actual usability was successfully developed. The process of avoidance of dual and toll bar obstacles clearly exposed the necessity of learning to allow collision-free trajectory and ETA driving. All collisions with obstacles occurred while blind participants were making a detour to avoid an obstacle, and about half of the collisions occurred between the body of blind participants and an obstacle. This means that the obstacle detection of the ultrasonic sensor and participants’ obstacle perception worked normally, but participants were not able to make an appropriate collision-free trajectory. The other collisions occurred between the ETA and an obstacle, which could be described as being for the same reason discussed above. Walking speeds of the blind participants are shown in Table 4. The average speed was about 0.863 km/h. This is not slow, especially considering that the blind participants attempted to avoid obstacles with no experience with the developed ETA.

To consider potential risk factors on the walkway, a hanging obstacle was added in the third walking test. There were many instances of successful obstacle avoidance in the third walking test, no less than achieved in the second walking test. There was only one collision with a single obstacle, and the dual and toll bar obstacles achieved 60% and 86.7% avoidance, respectively, as shown in Table 5. The avoidance rate of dual obstacles was relatively low, the same as in the second walking test. Considering that the walking test was performed without any prior learning and the degree of difficulty of dual obstacles, the avoidance rate of 60% is not so low an achievement. If an appropriate learning procedure for obstacle avoidance with the ETA is given to the blind user, it is expected that the avoidance rate will be increased. However, the successful avoidance of the hanging obstacle was much less. Only subject five succeeded twice. One reason for the low avoidance was that the hanging obstacle used in the test was a relatively small object (24 cm diameter ball). Although most participants did not succeed, many of the participants perceived the position of the obstacle and tried to avoid it. It was observed that participants who failed at the task gave up trying to avoid the obstacle. After the walking test, they gave the reason that the vibrotactile stimuli disappeared during the avoidance process. This means that it is necessary to adjust the sensors for hanging obstacle detection and to provide learning time for the developed ETA. The average walking speed jumped to about 1 km/h in the third walking test (as shown in Table 6), because the number of obstacles decreased.

To verify the detection of ground drop-offs, the braking test was performed, the results of which are shown in Table 7. The margin between the front wheel and ground drop-off was about 15 cm in the vertical approach to drop-off and about 10 cm in the 45 degree approach to drop-off. The approach speed of the ETA was set to 3 km/h, according to the walking speed of blind people. The test of the ETA braking system was performed under conditions that closely resembled the real approach of a ground drop-off. Therefore, the results from the braking tests show that the ETA with the ultrasonic sensor array can ensure safety from ground drop-offs. From the results of the first, second, and third walking tests as well as the braking tests, it was confirmed that the ETA with ultrasonic sensor arrays and tactile display is feasible.

Through the walking tests by the blind subjects and the safety evaluation of ground drop-offs, the feasibility of the developed ETA was verified. According to the survey of the developed ETAs for the blind [16,17] and the related works [1,2,3,4,5,6,7,8,9,10,11,12,13,14,15], it is hard to find a satisfactory ETA for the blind, especially in the feature of reliability and performance. In this respect, the practical test carried out in this work by the blind subjects under realistic outdoor environmental conditions, including the safety evaluation to down-step. Furthermore, the avoidance rate for single and toll bar obstacles was sufficiently high to use in daily life, and the other avoidance rates could be improved by providing an appropriate learning procedure with the developed ETA.

**Figure 15:**
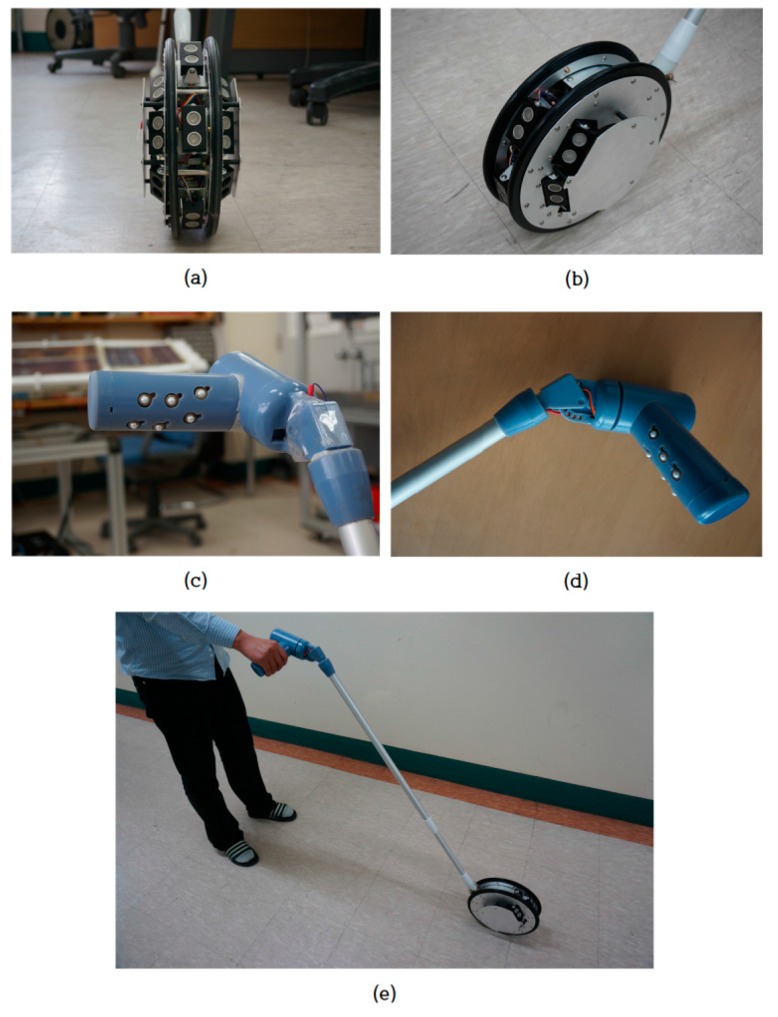
Assembled light-weight ETA with improved tactile display. (**a**) Front view; seven ultrasonic sensors are attached (2 × 3 for front and one for downward detection); (**b**) Side view; bearing-supported outer rim is rollable and the mechanism protects the sensors from an unexpected collision; (**c**) Implemented tactile display with 2 × 3 vibrotactile actuator to stimulate the corresponding fingers; (**d**) Grip-type tactile display enhances close contact of the user’s hand, and is adjustable to the posture of user’s hand; (**e**) User and the assembled ETA.

For improving portability and usability, the light-weight ETA was designed and assembled based on the evaluation of the developed ETA, as shown in Figure 15. The specification of the new ETA is shown in Table 8, and most of the function is the same as the developed ETA. The weight is sufficiently portable to handle and control in daily life, and the cost is low because the system consists of sonars, vibrators, microprocessors, and the wheeled cane. The ergonomically-designed grip-type tactile display fits to the hand of the user so that the display keeps close contact between the actuator and the finger parts to transfer the tactile stimulation efficiently. For the weight reduction of the ETA, the electromagnetic brake was removed. The detected ground drop-offs are notified by the activation of all tactile elements to stop walking and avoid the down steps. The light-weight structure gives sufficient portability in almost environments of daily life, and the ergonomically designed tactile display provides robust tactile feedback of obstacle information. The developed device is easy to use, and the interface between the user and the system is natural, so there is no need for an extensive training period.

Table 9 shows the comparison of the representative ETAs developed for visually-impaired persons. Most of the ETAs were designed for detecting or scanning of the walking path or front space only, so it was difficult to detect overhanging objects and/or down-steps. Additionally, most of the test was carried out in indoor environments by the blindfolded persons. The test by the blind users in the outdoor environment with realistic obstacles is a necessary step for validation of the feasibility of the developed ETA and for receiving feedback for future improvements. Some of the ETAs also use audio feedback to interface with the user. Despite the advantage of echolocation and spatial sound, the audio feedback interferes or blocks listening environmental sound including essential sign.

## 5. Conclusions

The goal of this study was to develop an easy-to-use ETA having the function of reliable object detection and its successful feedback to the user by tactile stimulation. An ETA including multiple ultrasonic sensors, electromagnetic brakes, and a tactile display was developed. Seven ultrasonic sensors were used to detect obstacles in the walking path. The sensors were arranged in a three by three array. The first and second rows of sensors in the array detect obstacle distribution on the ground, while the one sensor included in the third row detects ground drop-offs by monitoring ground level. To provide a high accordance between the position of the detected obstacle and the location of the perceived obstacle, tactile stimulators were designed with nine vibration actuators for stimulating the area of the fingers consisting of nine phalanges.

Walking tests were performed with visually-impaired participants using the developed ETA to verify the feasibility of the developed ETA in the outdoor environment. Each walking test was performed three times, and obstacle avoidance, walking speed with the ETA, and blind participants’ comments were evaluated. Evaluation of the braking system to ensure safety from ground drop-offs was also carried out. From the experiment, it was demonstrated that the feasibility of the developed ETA would be sufficient if the sensor ranges for hanging obstacle detection is improved and learning time for the ETA is provided.

Finally, the light-weight ETA designed and assembled based on the evaluation of the developed ETA was introduced. The reduced weight is sufficiently portable in the use of daily life, and the improved tactile display keeps close contact between the actuator and the finger parts to transfer the tactile stimulation efficiently. The comparison of the previously developed ETAs was also summarized and discussed.

## Figures and Tables

**Table 1:** Duty rate of vibration motor according to obstacle distance (OD).

	First Column	Second Column	Third Column
First Row	Duty rate (50%): 150 cm ≥ OD > 100 cm Duty rate (70%): 100 cm ≥ OD > 75 cm Duty rate (100%): 75 cm ≥ OD	Duty rate (50%): 200 cm ≥ OD > 150 cm Duty rate (70%): 150 cm ≥ OD > 100 cm Duty rate (100%): 100 cm ≥ OD	Duty rate (50%): 150 cm ≥ OD > 100 cm Duty rate (70%): 100 cm ≥ OD > 75 cm Duty rate (100%): 75 cm ≥ OD
Second Row	Duty rate (50%): 120 cm ≥ OD > 90 cm Duty rate (70%): 90 cm ≥ OD > 60 cm Duty rate (100%): 60 cm ≥ OD	Duty rate (50%): 200 cm ≥ OD > 150 cm Duty rate (70%): 150 cm ≥ OD > 100 cm Duty rate (100%): 100 cm > OD	Duty rate (50%): 120 cm ≥ OD > 90 cm Duty rate (70%): 90 cm ≥ OD > 60 cm Duty rate (100%): 60 cm ≥ OD

**Table 2:** Results of the first walking test.

	First Try (Success/Failure)	Second Try (Success/Failure)
Subject 1	10/0	10/0
Subject 2	9/1	9/1
Subject 3	9/1	10/0
Subject 4	9/1	10/0

**Table 3:** Results of the second walking test (failure of obstacle avoidance).

	First Try	Second Try	Third Try
Subject 1	1 (dual)	1 (dual)	1 (toll)
Subject 2	0	1 (dual)	0
Subject 3	0	1 (toll)	1 (dual)
Subject 4	2 (toll, dual)	1 (toll)	1 (toll)
Subject 5	2 (toll, dual)	0	0

Successful avoidance of Single obstacle: 100%; Dual obstacle: 60%; Toll bar: 60%.

**Table 4:** Results of the second walking test (time and speed).

	First Try	Second Try	Third Try
Subject 1	160 s (0.68 km/h)	159 s (0.68 km/h)	200 s (0.54 km/h)
Subject 2	93 s (1.16 km/h)	115 s (0.94 km/h)	120 s (0.90 km/h)
Subject 3	113 s (0.96 km/h)	119 s (0.91 km/h)	109 s (0.99 km/h))
Subject 4	156 s (0.69 km/h)	121 s (0.89 km/h)	110 s (0.98 km/h)
Subject 5	142 s (0.76 km/h)	121 s (0.89 km/h)	110 s (0.98 km/h)

Average speed: 0.863 km/h.

**Table 5:** Results of the third walking test (failure of obstacle avoidance).

	First Try	Second Try	Third Try
Subject 1	1 (hang)	1 (hang)	2 (hang, toll)
Subject 2	1 (hang)	2 (hang, dual)	1 (hang)
Subject 3	2 (single, hang)	3 (dual, toll, hang)	2 (dual, hang)
Subject 4	2 (dual, hang)	1 (hang)	2 (dual, hang)
Subject 5	0	1 (dual)	1 (hang)

Successful avoidance of Single obstacle: 96.7%; Dual obstacle: 60%; Toll bar: 86.7%; Hanging obstacle: 13.3% *. (* perception only: 66.7%)

**Table 6:** Results of the third walking test (time and speed).

	First Try	Second Try	Third Try
Subject 1	137 s (0.79 km/h)	132 s (0.82 km/h)	119 s (0.91 km/h)
Subject 2	113 s (0.96 km/h)	111 s (0.92 km/h)	90 s (1.20 km/h)
Subject 3	126 s (0.86 km/h)	140 s (0.77 km/h)	98 s (1.10 km/h)
Subject 4	128 s (0.84 km/h)	98 s (1.10 km/h)	90 s (1.20 km/h)
Subject 5	109 s (0.99 km/h)	79 s (1.37 km/h)	90 s (1.20 km/h)

Average speed: 1.002 km/h.

**Table 7:** Results of braking system test (unit: cm).

	First	Second	Third	Fourth	Fifth	AVG (STD)
90°	14.5	15.5	14.5	15	15	14.9 (0.42)
45°	9.5	10.5	10	9.5	9.5	9.8 (0.45)

**Table 8:** Specification of light-weight ETA.

Total Weight	1.75 kg	
Diameter of wheel	248 mm	Rollable and sensor protect mechanism
Length of rod	970 mm	Adjustable to user’s height
Control unit	2 × ATmega128	Signal processing and actuator control
Sensing unit	7 ultrasonic sensors	Front obstacles and down-step detection
Tactile display	2 × 3 vibrotctile actuator	Replaceable to the need
Running time	5 h	In case of full operation (Li-Po/2600 mAh)

**Table 9:** Comparison of developed ETA for visually-impaired persons.

	Features	Detecting Range	Test		Feedback	Portability	Cost	Limitations
System			Participant	Task & Environment				
Navbelt [9]		Walking path	Blindfolded	Obstacle avoidance while indoor/outdoor walking	Audio	Portable	Medium	- Limited detection ability Bulky prototype - Required extensive training
vOICe [12]		Front space	Blindfolded	Operation and feasibility of the system	Audio	Portable	Low	- Required extensive training - Not yet tested on blind users
NAVI [13]		Front space	Blind	Object identification in indoor environment	Audio	Portable	Medium	- No information about the distances of objects
GuideCane [10]		Walking path	Blind/Blindfolded	Navigating corridors/Traversing cluttered indoor area	Vibrotactile	Portable	Medium	- Limited scanning area - Bulky prototype
ENVS [14]		Walking path	Blindfolded	Obstacle avoidance while outdoor walking	Electrotactile	Wearable with portable computer	Medium	- Difficult to detect ground or overhanging objects
EPFL [11]		Walking path	Blindfolded	Navigating through a corridor	Vibrotactile	Wearable	Low	- Difficult to detect ground objects - Interference of hands to detect obstacles
Tyflos [15,16]		Front space	Blindfolded	Operation of the system in indoor area	Vibrotactile/Audio (optional)	Wearable with portable computer	Medium	- Not yet tested on blind users - Difficult to detect down-steps
EyeCane [23]		Points scanning by user	Blind/Blindfolded	Obstacle avoidance and navigation in mazes	Audio/Vibrotactile	Hand-held	Low	- Needs continuous detecting effort
Proposed system (CBNU ETA)		- Walking path - Overhang - Down step	Five blind persons	Avoidance of 5–7 obstacles while outdoor walking	Vibrotactile	Portable	Low	- Low avoidance rate for overhanging object
